# Adult lung stem cells and their contribution to lung tumourigenesis

**DOI:** 10.1098/rsob.120094

**Published:** 2012-08

**Authors:** Marie-Liesse Asselin-Labat, Caitlin E. Filby

**Affiliations:** 1 ACRF Stem Cells and Cancer Division, The Walter and Eliza Hall Institute of Medical Research, Parkville, Victoria, Australia; 2 Department of Medical Biology, The University of Melbourne, Parkville, Victoria, Australia

**Keywords:** lung stem cells, lung disease and repair, lung cancer, cell of origin of lung cancer, cancer stem cells

## Abstract

The isolation and characterization of lung stem and progenitor cells represent an important step towards the understanding of lung repair after injury, lung disease pathogenesis and the identification of the target cells of transformation in lung carcinogenesis. Different approaches using prospective isolation of progenitor cells by flow cytometry or lineage-tracing experiments in mouse models of lung injury have led to the identification of distinct progenitor subpopulations in different morphological regions of the adult lung. Genetically defined mouse models of lung cancer are offering new perspectives on the cells of origin of different subtypes of lung cancer. These mouse models pave the way to further investigate human lung progenitor cells at the origin of lung cancers, as well as to define the nature of the lung cancer stem cells. It will be critical to establish the link between oncogenic driver mutations recently discovered in lung cancers, target cells of transformation and subtypes of lung cancers to enable better stratification of patients for improved therapeutic strategies.

## 2. Introduction

Respiratory diseases are a major cause of mortality and morbidity worldwide, with over 10 million deaths attributed to lung disorders [1]. The lung is a complex organ with multiple functions that are critical for survival. Isolation and characterization of lung stem cells and understanding their capacity for repair, regeneration and tumourigenesis have an enormous potential impact on prevention and treatment of lung diseases. Lung stem cells may constitute a therapeutic option in poorly treated lung degenerative disorders, including cystic fibrosis, chronic obstructive pulmonary disease and idiopathic pulmonary fibrosis. Comprehension of the epithelial hierarchical organization of the normal lung is also critical for the understanding of the initiation of lung carcinogenesis. Recent advances in the technologies available for cell tracking using engineered mouse models, as well as cell isolation by flow cytometry, have provided new tools to study lung stem/progenitor cells. In this review, we focus our interest on recent insights into the identification of lung stem and progenitor cells in the adult lung, and the evaluation of their role as possible cells of origin in lung cancer.

Lung cancer is the leading cause of cancer death worldwide. Five-year lung cancer survival is only 15 per cent [2], and lung cancer is responsible for more deaths than prostate, colon, pancreas and breast cancers combined. Major improvements in clinical outcome will depend on new insights into normal lung and tumour biology. Lung cancers are divided into distinct histopathological classes: small cell lung cancer (SCLC; 20% of all lung cancers), which has a neuroendocrine phenotype, and non-small cell lung cancer (NSCLC; 80% of all lung cancers), which can be further subdivided into adenocarcinomas, squamous cell, bronchioalveolar and large cell carcinomas [3–6]. Squamous cell carcinomas are thought to originate from the proximal airways, SCLC are predominantly located in the bronchioles while adenocarcinomas, the most common type of lung cancer, are more frequently detected in the distal part of the lung. It is speculated that these different subclasses arise from distinct cells of origin localized within a defined regional compartment [7,8]. Prospective isolation of stem/progenitor cells in the different compartments of the lung will enable further evaluation of their respective roles in tumour initiation.

A large number of cell types constitute the adult lung and are present at different frequencies according to the anatomical region of the respiratory system [9]. In the adult trachea and main bronchi (cartilaginous airways), the luminal epithelium contains two main columnar cell types: ciliated cells (expressing FoxJ1) and Clara-like cells (producing secretoglobins, the most abundant being Scgb1a1, or CC10). Ciliated cells are terminally differentiated cells that do not have self-renewal capacity [10,11]. A small number of neuroendocrine cells are also present. The cartilaginous airways contain a discontinuous population of basal cells that express p63, keratin 5, keratin 14 and nerve growth factor receptor (NGFR) [12]. In the mouse, basal cells are only detected in the trachea, whereas in humans basal cells are present in the bronchi and bronchioles [12]. In the more distal airways (small bronchi and bronchioles), the epithelium is columnar. Clara cells predominate over ciliated cells and there are more neuroendocrine cells than in the trachea. No basal cells are detected in the distal small airways [13,14]. The most distal region of the lung is organized into a complex system of alveoli composed of two types of epithelial cells: alveolar type I cells (AEC I), which provide the thin-walled gas exchange surface, and cuboidal alveolar type II cells (AEC II), containing secretory vesicles filled with surfactant, including surfactant protein C (SP-C). The transitional region between the terminal bronchiole and the alveoli is known as the bronchioalveolar duct junction [15]. These different regions of the lung appear to use different progenitor cells for maintenance and repair [15].

## 3. The importance of models of lung injury to study lung stem cells

**Figure 1.**
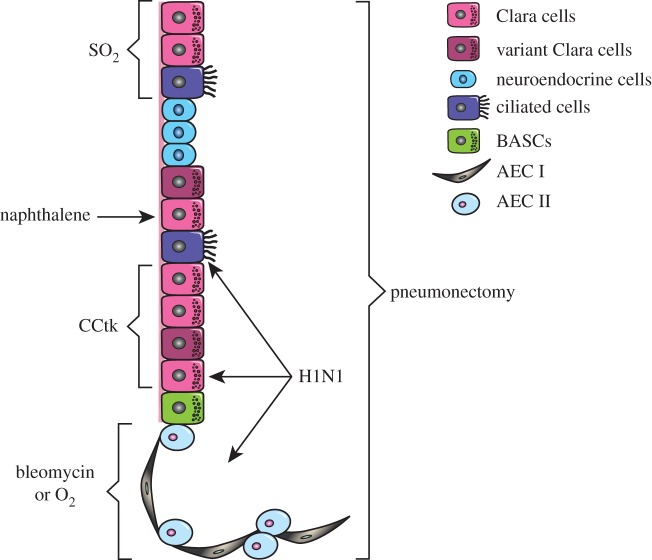
Models of lung injury to study lung stem cells. Schematic diagram of the selective effect of different injuries in proximal and distal lung.

Different models have been proposed for the maintenance and regeneration of adult solid tissues. In breast and gut, a small number of undifferentiated stem cells can self-renew and produce differentiating progeny for normal tissue function [16–18]. In the skin, Clayton *et al.* [19] proposed a committed progenitor model in which the epidermis is maintained by a population of progenitor cells that can undergo unlimited cell divisions and terminal differentiation [20,21]. Other organs (such as the pancreas and the liver) seem to regenerate by simple proliferation of existing mature cells such as β-cells or hepatocytes, but can also use ‘facultative’ stem cells to regenerate the tissue [22–26]. The model followed by the lung epithelium at steady state and after injury is still a matter of debate. Compared with the intestine or the skin, the adult lung has a slow turnover time. It is constantly exposed to potential toxic agents and pathogens present in the environment, however, and must therefore be able to respond quickly and effectively to cellular damage, suggesting the existence of lung stem/progenitor cells. Myelo-ablation and competitive repopulation assay have been used for many years in the haematopoietic field to study haematopoietic stem cell activity [27]. Similarly, in the lung, several experimental protocols (described below, and summarized in table 1 and figure 1) have been developed in mice to challenge the lung and stimulate activation of stem/progenitor cells [15,40]. Each model is unique in the injury caused, the degree of immune cell infiltration and fibrosis, the cell types affected, and resulting regeneration. In-depth description of lung injury models have been reviewed elsewhere [15,40]. Here, we describe mouse models most recently used in the search for adult lung stem cells (table 1 and figure 1).

**Table 1.** Models of lung injury to study lung stem cells.

model	dose and route of administration	target cell(s)	maximal injury	repair	references
bleomycin	2.3 units kg^–1^ intratracheal instillation or, 120 mg kg^–1^ i.v.	EC, AEC I, AEC II	6–10 days	21 days	[28–30]
naphthalene	250 mg kg^–1^, i.p.	Cyp2f2-containing Clara cells	3 days	10 days	[31,32]
ganciclovir (CCtk mice)	4.5 mg d^–1^ GCV (375 mg ml^–1^ in saline) via miniosmotic pump for 6 or 12 days	CC10^+^ Clara cells, ciliated cells susceptible to delayed ablation, AEC II	7–12 days		[33–35]
pneumonectomy	n.a.; entire left lung removed	all lung epithelia, vasculature and support cells	at time of surgery	15 days	[36]
H1N1 (PR8) influenza virus	250 PFU, intratracheal inhalation	Clara cells, ciliated cells, AEC II	11 days	21–60 days	[13]
O_2_	70–100% O_2_, chamber for 56 h or if longer, alternating to room air every 24 h	alveolar cells (distal)	3 days	14 days	[37,38]
SO_2_	500 ppm SO_2_ in room air, chamber for 3 h	luminal cells of tracheo-bronchial epithelium	36 h	7 days	[11,14,39]

### 3.1. Naphthalene

Naphthalene is an aromatic hydrocarbon found in tobacco smoke and in mothballs. Administered i.p. naphthalene becomes cytotoxic when metabolized by Cyp2f2, a specific P450 mitochondrial cytochrome contained in a subset of Clara cells located in the bronchioles [31,32]. Approximately 3 days after naphthalene administration, the majority of Clara cells lining the bronchioles are destroyed. This effect is abolished in mice lacking Cyp2f2 [31]. A small subset of Clara cells, termed variant Clara cells, are resistant to naphthalene and are proposed to be responsible for repletion of the bronchiolar epithelium after injury [31,32,41].

### 3.2. Ganciclovir (CCtk mice)

To target all Clara cells independent of Cyp2f2 expression, Reynolds *et al.* [33] generated a transgenic mouse strain, termed CCtk, which possess the herpes simplex virus thymidine kinase (HSVtk) under the control of the CC10 promoter. Temporal and site-specific ablation is achieved by the addition of ganciclovir, which results in production of toxic HSVtk metabolites in cells expressing HSVtk, in this case Clara cells [33]. Whereas variant Clara cells are resistant to naphthalene, the CCtk mouse model results in complete depletion of CC10^+^ cells, making it a useful model to identify early Clara cell, progenitors. Secondary loss of AEC II was observed in these mice and was characteristic of an end-stage disease [34].

### 3.3. Bleomycin

Bleomycin is an antibiotic produced by *Streptomycin vercillus* that has been used extensively as anti-cancer agent owing to its ability to cause DNA strand breaks. A major side effect of the drug is pulmonary fibrosis, specifically bronchioalveolar damage. In mice, reduction in the number of AEC I and AEC II was observed after intranasal or intratracheal instillation [28,42,43]. Intratracheal administration, the most frequently used method, results in maximum AEC I and AEC II loss 6–10 days following treatment [29,30,44,45].

### 3.4. Pneumonectomy

Partial pneumonectomy (PNX), whereby one lobe is removed by surgical resection, results in compensatory expansion of the remaining lung lobes, which increase in volume to fill the void and maintain ventilation [46–48]. Recently, Ding *et al.* [36] provided new insight into this long-standing yet unexplored model of lung regeneration. Use of PNX demonstrates the interplay between endothelial and epithelial compartments, long known to be essential for developmental alveologenesis, but only recently appreciated as critical for regenerative alveologenesis [36].

### 3.5. H1N1

Sublethal infection of mice with murine adapted (PR8) H1N1 influenza virus results in widespread bronchiolar and alveolar damage, with loss of Clara cells, ciliated cells and AEC II. Remarkably, post-H1N1 lung regeneration appears to occur in the absence of fibrosis [13]. The infiltration of macrophages appears essential for the post-H1N1 regenerative process to occur without fibrosis [49].

### 3.6. O_2_ and SO_2_

The diffusible gases O_2_ and SO_2_ are administered via ventilation supply of the gases to mice housed in airtight chambers, and result in lung injury with immune cell infiltration and fibrosis. Hyperoxia has long been known to cause lung injury and changes in normal alveolar development in premature infants on artificial ventilation. In adult mice, O_2_-induced hyperoxia causes alveolar epithelial cell death by day 3 (reviewed in [37]) and repair is complete by day 14. The primary phase of hyperoxic lung toxicity consists of damage to the epithelium and endothelium that results in oedema and immune cell infiltration. The subsequent secondary phase consists of proliferation of AEC II, interstitial fibrosis and impaired gas exchange (reviewed in [50]). Inhaled SO_2_ causes destruction of the luminal cells of the pseudo-stratified tracheo-bronchial epithelium [11,51], the distal lung epithelium being spared [39].

Other agents that cause lung injury include nitric oxide, ozone, chlorine, polidocanol and particulates, among others [52–54]. Future work that determines *in vivo* the specific cells affected and those responsible for the repair to injury in these injury models may provide further tools to investigate adult lung stem cells.

## 4. The search for adult lung stem cells

Use of the murine models of lung injury described above has enlightened our understanding of lung regeneration and led to the identification of lung stem/progenitor cells. It is becoming evident that in mice diverse types of injury activate different signalling pathways, leading to the activation of different types of progenitor cells, and that different regions of the respiratory system (alveoli, bronchioles and upper airways, i.e. bronchi and trachea) have different kinds of progenitor cells for maintenance and repair [15].

Regeneration of the lung parenchyma after injury is thought to be dependent on SP-C^+^ AEC II cells that can proliferate and regenerate AEC I cells after injury [43,55,56]. Recent lineage tracing experiments in mice unequivocally showed that at steady state and in response to bleomycin injury, AEC I cells were generated from AEC II cells [45]. However, newly generated AEC II cells after injury were derived from SP-C^−^ cells, suggesting the existence of an SP-C^−^ alveolar progenitor population capable of regenerating the AEC II cells in the injured distal lung [29,45]. Chapman *et al.* [29] recently identified a population of CD49f^+^CD104^+^ (integrin-α6^+^β4^+^) alveolar epithelial cells in the murine distal lung enriched for SPC^−^CC10^−^ cells. These cells have the capacity to give rise to SP-C^+^ and CC10^+^ cells *in vitro* or after transplantation under the kidney capsule when aggregated with embryonic lung cells. These progenitor cells may therefore be the precursors of differentiated AEC II SP-C^+^ cells [29]. Using additional cell surface markers, McQualter *et al.* [57] isolated three distinct subpopulations of mouse lung epithelial cells and evaluated their colony formation capacity *in vitro*. The EpCAM^hi^CD104^+^CD24^lo^ subset is enriched in cells with colony-forming capacity, capable of self-renewal and forming colonies composed of airway, alveolar or mixed lung epithelial lineages *in vitro.* They may be similar to CD49f^+^CD104^+^ cells identified by Chapman *et al.* [29]. The EpCAM^med^CD104^−^ subset is enriched in alveolar cells and only a small fraction of these cells has alveolar-committed progenitor activity with the generation of saccular, AEC II-like colonies, whereas EpCAM^hi^CD104^+^CD24^hi^ cells did not exhibit colony-forming capacity and were enriched in ciliated cells [57].

In the terminal bronchiole, cells located at the bronchio-alveolar ductal junction were proposed to be responsible for repair after injury in mice. Slow-cycling label-retaining cells expressing the Clara cell-specific marker CC10 were observed after naphthalene injury [58]. Subsequently, a population of putative bronchio-alveolar stem cells (BASCs) resistant to bronchiolar and alveolar damage was described [30]. These cells coexpressed CC10 and SP-C and expanded subtly after bronchiolar (naphthalene-induced) or alveolar (bleomycin-induced) injury. *In vitro*, BASCs had self-renewal capacity and when cultured on Matrigel could differentiate into Clara cells, AEC I and AEC II cells, but did not differentiate into ciliated cells. Cell surface markers to isolate the BASCs are still a controversial and unresolved question. Initial studies demonstrated an enrichment of BASCs in Sca-1^+^CD34^+^ cells [30]. But Teisanu *et al.* proposed that bronchiolar progenitor cells, resistant to naphthalene injury, were enriched in the Sca-1^lo^CD34^−^ subset and could be further separated from Clara cells based on their level of autofluorescence [59,60]. McQualter *et al.* demonstrated that mouse lung Sca-1^+^ cells were enriched in fibroblasts that could support the growth of epithelial progenitor cells, further indicating that Sca-1 is probably not a marker of epithelial progenitor cells [57,61]. Further refinement of the cell surface markers expressed by BASCs suggested that they were enriched in the EpCAM^hi^CD104^+^Sca-1^lo^CD24^lo^ subset [62]. These discrepancies in the cell surface markers described by different groups may be the result of distinct tissue processing, as well as analytical approaches. However, there is accumulating evidence to propose that the EpCAM^hi^CD104^+^CD49f^+^Sca-1^lo^CD24^lo^ subset is enriched in mouse lung progenitor cells [29,57,61,62], but whether these cells express SP-C and/or CC10 is still unresolved. Combining cell surface marker studies with lineage tracing experiments using a split-Cre approach, in which inactive ‘split-Cre’ fragments are controlled by two different promoters (e.g. N-cre controlled by SP-C promoter and C-cre controlled by CC10 promoter) and regain Cre activity when overlapping expression exists, will help resolve this question [63].

In the bronchioles, Clara cells are capable of self-regeneration and generate terminally differentiated ciliated cells in mice. *In vivo* lineage tracing experiments using CC10-creER^Tam^ mice showed that CC10-expressing Clara cells in the bronchioles self-renewed and generated ciliated cells during post-natal growth, adult homoeostasis and repair after bronchiolar injury [41]. Interestingly, damage of the alveolar compartment by hyperoxia in CC10-creER^Tam^ mice did not yield to the production of lineage-labelled AEC I and AEC II cells, suggesting that in this model CC10^+^ Clara cells in the bronchiole could not generate alveolar cells [41]. In contrast, lineage-labelled AEC II and AEC I cells were detected in fibrotic regions after bleomycin-induced alveolar injury in CC10-creER^Tam^ mice [45]. Although it is still unclear whether the lineage-labelled AEC II and AEC I cells observed following bleomycin injury in CC10-creER^Tam^ mice are derived from BASCs, lineage-labelled AEC II or SP-C negative alveolar cells, it would be of interest to identify the signals mediated by bleomycin, but not by hyperoxia, that can induce differentiation of CC10^+^ cells into alveolar lineages.

In the upper airways, basal cells and not Clara cells were found to be the precursors of the tracheal lineages in mice. Lineage tracing of CC10-labelled cells during ontogeny in the trachea showed an initial increase in labelled ciliated cells followed by a decrease in the number of labelled Clara cells and ciliated cells over time. After SO_2_-induced tracheal injury, proliferation of CC10^+^ cells was observed, but the majority of the newly formed tracheal epithelium was unlabelled. These results suggested the existence of a CC10^−^ epithelial progenitor population responsible for maintenance of the tracheal luminal epithelium during post-natal growth, adult homeostasis and repair [41]. Lineage-tracing experiments of the basal cells using keratin 5-creER^Tam^ or keratin 14-creER^Tam^ strains showed that these cells in the mouse trachea have the potential to self-renew and generate both Clara cells and ciliated cells *in vivo* during post-natal growth and after injury, placing the basal cells at the apex of the cellular hierarchy to generate and repair the tracheal epithelium [14,64]. These cells were further isolated from the mouse trachea and human airways based on the expression of CD49f (integrin α6) and NGFR, and formed ‘tracheospheres’ or ‘bronchospheres’ in *in vitro* culture [14]. At steady state, basal cells are only detected in the trachea of the mouse, in contrast with the human airway epithelium where keratin-5^+^/keratin-14^+^ cells are also detected in the bronchi and bronchioles [12]. However, two mouse models of lung injury demonstrated the emergence of basal cells in the mouse bronchi and the distal lung, suggesting that basal cells could play a transient role in mouse distal lung regeneration after injury [65]. Proliferative keratin-14^+^ cells were detected 1 day following naphthalene injury in the mouse bronchi, and gave rise to Clara cells and ciliated cells [13,65]. Using a different mouse model of injury, Kumar *et al.* [13] recently described a population of p63^+^, keratin 5^+^ basal cells that appear after influenza A virus (H1N1) sub-lethal injury in the distal lung that was not detected after bleomycin injury. Gene expression profiling and lineage tracing experiments suggested that these cells participated in the restoration of the injured alveoli after H1N1 infection and expressed high levels of angiogenic factors to promote neo-capillary formation [13]. Kumar *et al*. proposed that the keratin-5^+^ cells observed after H1N1 injury originated from a rare population of basal cells present in the distal mouse lung. However, we (M.-L. Asselin-Labat 2012, unpublished data) and others [14] were not able to detect keratin-5 positive cells in the distal lung by immunohistochemistry. Lineage-tracing experiments may help define the origin of the basal cells observed after influenza-induced injury and define a population of early progenitor cells.

These studies highlight how different mouse models of lung injury have been used to identify progenitor cells in the lung. Each model activates different regenerative properties. Interestingly, chemical injury (bleomycin), hyperoxia and viral infection (H1N1), although all damaging the distal lung, appear to stimulate distinct signalling pathways, leading to the activation of different types of progenitor cells to induce lung regeneration. Comparison of those different signals would generate insights into the processes responsible for the activation of a specific progenitor cell type. A caveat of murine models of the lung is that there are well-described differences between mouse and human lung. These include the absence of respiratory bronchioles and a reduction in both the number of airway generations and submucosal glands in the mouse, as well as absence or limited number of basal cells in mouse airways compared with human airways [12,66]. Translating the results described in mice to the human lung is critical for understanding human lung biology and pathology. However, the search for human adult lung stem cells has proved a lot more difficult and only minor advancement has been made. Kajstura *et al.* recently published a controversial study [67,68] claiming the identification of human lung stem cells based on the expression of c-kit [69]. The most surprising finding in this work is the unprecedented identification of cells that can give rise to both endodermal and mesodermal lineages. Thorough replication of this work will be necessary to confirm their claim. The gold standard assay to assess stem cell property is *in vivo* repopulation after challenge of the environment to generate a stem cell niche. Developing such an assay in the lung constitutes a major challenge, but will be instrumental to demonstrate the existence of mouse and human lung stem cells. Combining mouse lineage tracing experiments with prospective isolation of lung stem cells with cell surface markers will enable further delineation of their molecular characteristics to identify genetic and epigenetic factors regulating their function in normal lung and diseased lung, including lung cancer.

## 5. Regulators of adult lung stem/progenitor cells

Pathways regulating embryonic lung development have been well studied (reviewed in [70]), but signalling pathways regulating cell proliferation, self-renewal or differentiation in the adult lung are still largely underexplored, in large part due to the paucity of markers available to prospectively isolate lung stem and progenitor cells. In the mouse lung subpopulations described earlier, the proportion of progenitor cells remains low, with only approximately 5 per cent of EpCAM^hi^CD104^+^CD24^lo^ cells having colony-forming capacity *in vitro* [57], while limiting dilution studies showed that 1 in 110 cells in the BASC-enriched population had colony-forming potential [71]. This presents a major limitation for the use of gene profiling studies to identify pathways regulating stem/progenitor cells.

Recent studies have relied on gain or loss of function of genes known to be regulators of self-renewal in other stem cell systems, such as Bmi-1, β-catenin or Notch. Bmi-1 regulates stem cell self-renewal and cancer progression in many organs, including the haematopoietic and neural systems and the breast [72–74]. Bmi-1 is also involved in chromatin remodelling and is upregulated during lung organogenesis, where it may play a role in enhancing the accessibility of transcription factor binding sites [75]. In Bmi-1-deficient mice, lung repair after naphthalene-injury was impaired [71]. Bmi-1-deficient BASCs were less proliferative than wild-type BASCs *in vitro* and failed to self-renew [71]. Loss of Bmi-1-target genes *p16/p19* only partially rescued the self-renewal capacity of Bmi1-deficient BASCs *in vitro*, whereas loss of the imprinted gene *p57* largely reactivated Bmi-1-deficient BASCs self-renewal capacity [62,71]. β-catenin, a downstream target of the Wnt pathway, regulates stem cell self-renewal [76,77]. Stabilization of β-catenin in Clara cells resulted in an accumulation of progenitor cells resistant to naphthalene injury, leading to increased cell proliferation and earlier lung repair [78]. The Notch pathway plays an important role in embryonic lung development to maintain the balance of proximal–distal cells at early stages and in cell fate decision later in development. In particular, Notch favours a non-neuroendocrine fate, and promotes mucous cell differentiation at the expense of ciliated cells of the conducting airways and alveolar cells of the distal airways [79–82]. In the adult, family members of the Notch signalling pathway are expressed in the basal epithelial cells of the adult mouse trachea [51]. Notch activation in the basal cells of the adult mouse trachea resulted in their differentiation into the secretory luminal lineage following SO_2_ injury [51]. Conversely, loss of Notch function resulted in a significant reduction in the number of luminal cells in SO_2_-injured trachea. Mouse basal cells treated with the γ-secretase inhibitor dibenzazepine (inhibitor of Notch signalling) led to the formation of p63^+^ tracheospheres that did not express luminal cell markers, indicating that although Notch is not required at steady state in the trachea, it is required for basal cell differentiation into luminal cells after tracheal injury [51].

Development of the embryonic lung is regulated by endodermal–mesenchymal cross-talk, and alveolarization of the embryonic lung is highly controlled by parallel blood vessel formation [70,83–85]. Similarly, in the adult lung, progenitor activity is tightly controlled by autocrine and paracrine signals released by other cells types. Co-culture of mouse lung epithelial progenitor cells with mouse lung fibroblasts was required to induce multi-lineage differentiation of the epithelial cells *in vitro* [57]. Recent evidence showed the importance of interactions between the lung epithelium and the vasculature for adult mouse lung regeneration [36]. Stimulation of pulmonary capillary endothelial cells after PNX led to the production of angiocrine factors, including MMP14 in a VEGFR2/FGFR1-dependent manner. Expression of VEGFR2 and FGFR1 in the endothelium was required to stimulate pulmonary capillary endothelial cells to support neo-angiogenesis. This resulted in expansion of BASCs and AEC II amplification, as demonstrated by the use of endothelium-specific *vegfr2/fgfr1* deletion in mice [36]. Similarly, genes involved in angiogenesis and endothelin signalling were detected in regions of lung repair after H1N1 infection [13], further indicating that endothelial–epithelial interactions are involved in repair of the lung in response to varied injurious stimuli.

## 6. Cells of origin in lung cancer

Identifying distinct populations of stem or progenitor cells in the lung has important implications for a better understanding of normal lung function and lung disease processes [9]. It is also key to better understand lung cancer pathology, and to determine the cell of origin of different subtypes of lung cancer [86]. The target cell of transformation for most cancers is unknown. Although there is evidence that certain types of leukaemia arise from mutations that accumulate in haematopoietic stem cells, more recent work suggests that the cell of origin of acute myeloid leukaemia or the basal type of breast cancer may reside in committed progenitor rather than stem cell populations [87,88]. Gene profiling studies of lung cancers led to further stratification of the histopathological subtypes of lung cancer into distinct molecular subgroups [5,89,90]. This heterogeneity probably reflects different oncogenic transformations occurring in different cell types. Until now, insights into the cell of origin have come from genetically defined mouse models of lung cancer.

**Figure 2.**
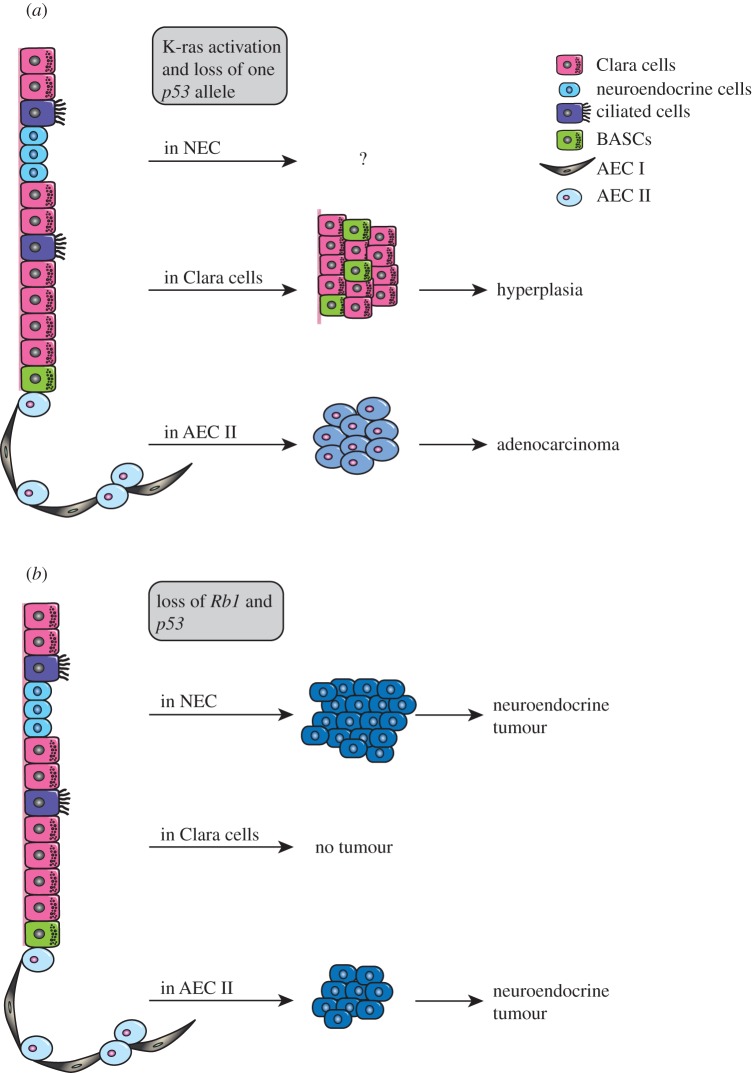
Cell of origin of lung cancers. (*a*) Models for adenocarcinoma formation in K-ras^G12D^p53^f/+^ mice. Alveolar epithelial type II cells (AEC II) are the most probable cells of origin of adenocarcinoma in these mice. (*b*) Models for SCLC formation in Rb1^f/f^;p53^f/f^ mice. Neuroendocrine cells are the most probable cells of origin of neuroendocrine tumour in this mouse model.

K-ras is mutated in 15 to 20 per cent of NSCLC [91]. Mouse models with oncogenic K-ras (K-ras^G12D^) expression have been developed to mimic human lung cancer [92]. Conditional expression of oncogenic K-ras^G12D^ after intratracheal or intranasal administration of adenovirus-cre in mice results in the formation of lung adenocarcinoma [93–95]. Additional *p53* mutation accelerates tumour formation and increases the metastatic properties of tumour cells, making it a mouse model more similar to human advanced lung adenocarcinoma [92,96]. These models have been successfully used to predict response to treatment [97], but also to study the cells of origin in lung adenocarcinoma. Treatment of mice with naphthalene, a component of cigarette smoke, accelerated tumour formation in K-ras^G12D^ mice, and expansion of BASCs was reported in K-ras^G12D^ mice [30]. These results and the observations that BASCs are expanded after naphthalene injury suggested that CC10^+^SP-C^+^ BASCs may be the cell of origin of K-ras^G12D^-driven lung adenocarcinomas. However, more recent work in which activation of the oncogenic K-ras and loss of one allele of *p53* was specified to CC10^+^ cells resulted in hyperplasia at the bronchio-alveolar ductal junction that did not evolve to adenocarcinoma [98] (figure 2*a*). Conversely, K-ras^G12D^ activation and *p53* heterozygosity in SP-C^+^ AEC II cells led to the formation of adenocarcinoma in the alveolar region of the lung. The survival of SP-C-cre;K-ras^G12D^p53^f/+^ mice was reduced by 10 weeks compared with CC10-cre;K-ras^G12D^p53^f/+^ mice, suggesting that SP-C^+^ cells may be the cells of origin in these genetically defined tumours [98]. Activation of K-ras^G12D^ exclusively in SP-C^+^CC10^+^ putative BASCs will help to resolve the role of this rare population in K-ras-driven lung cancers. Interestingly, K-ras-mutated lung adenocarcinomas in humans seem to be more prevalent in the distal lung than the proximal airways [7], further suggesting that AEC II cells may play an important role in the initiation of this subtype of lung tumours. Using a similar approach of specific activation of K-ras^G12D^ in other lung epithelial cell types such as the neuroendocrine cells may help further understand the connection between the cell of origin, oncogenic mutation and subtypes of lung cancers.

SCLCs have the phenotypic characteristics of neuroendocrine tumours, expressing neural cell adhesion molecule, synaptophysin and calcitonin gene-related peptide (CGRP). *Rb1* and *p53* loss of heterozygosity or mutations are present in 70 per cent of SCLCs [91]. This double inactivating mutation was reproduced in mice to generate a mouse model of SCLC that recapitulated the human phenotype [99]. Naphthalene injury did not accelerate tumour burden in this SCLC model [100]. To evaluate the cell of origin of SCLC, inactivation of *Rb1* and *p53* in different cellular compartments of the lung using cell-specific promoter (CC10-cre, SP-C-cre and CGRP-cre) adenoviral cre administration in Rb1^f/f^;p53^f/f^ mice was performed [100] (figure 2*b*). These experiments showed that neuroendocrine cells were the most probable cell of origin of SCLC. CGRP-cre-driven *Rb1/p53* loss resulted in tumour development in all animals, with a median tumour latency of a year. Interestingly, SP-C-cre driven deletion of *Rb1/p53* resulted in neuroendocrine tumour with the same phenotype as CGRP-cre-driven tumours in half of the animals, but with an extended median tumour latency [100]. Inactivation of *Rb1* and *p53* in CC10-positive Clara cells only yielded rare tumours in animals over 18 months old, indicating that Clara cells do not contribute to SCLC formation in this model (figure 2*b*).

In these genetically defined models (K-ras^G12D^/p53 and Rb1/p53), Clara cells do not appear to be the cells of origin of lung tumours. It remains to be explored whether any other genetic changes occurring in Clara cells would lead to cell transformation and the appearance of a distinct subtype of lung cancer, or whether Clara cells, although playing a role in lung repair after injury, may play a limited role as the cells of origin of lung cancers. The role of basal cells as the cell of origin of lung tumour was not evaluated in these models. Given that basal cells appear to sit at the top of the mouse lung epithelial hierarchy in the trachea [14] and are activated in the distal lung after injury [13,65], their role in lung tumourigenesis would be worthy of further investigation. Such work has been hampered due to the lack of specificity of basal cell markers in the upper airways (p63, Keratin-5, Keratin-14). These markers are also expressed in other epithelial organs, including the skin, limiting their use to induce oncogenic transformation in the lung. Identification of lung basal cell-specific markers will be necessary to evaluate their role in lung tumour initiation. *In vivo* cell-specific activation of oncogenes or inactivating mutation of tumour suppressor genes can only be performed in mouse models. Other approaches will be required to decipher the cell of origin in the different subtypes of human lung cancers. One question that remains is this: what are the phenotypic characteristics of the cells acquiring the first oncogenic transformation in these mouse models? How do they relate to stem/progenitor cells described above? Identification of the cell surface markers expressed by these different cell types will enable identification of key factors driving their proliferation. These cell surface markers may also be translated to human lung tumours and provide insights into the cell of origin of human lung cancers.

## 7. Cancer stem cells

The cancer stem cell model is based on the hypothesis that tumours are organized in a hierarchical way, and only a small proportion of cells with stem-like properties has the capacity to propagate the tumour and generate the different cell types constituting the tumour [101]. The origin of the cancer stem cell is not necessarily the normal stem cell but could be a committed progenitor cell that reverts to a stem-like phenotype during transformation. Evidence exists in the literature that leukaemia as well as some solid tumours may follow a cancer stem cell model while other tumour types such as melanoma follow the clonal evolution model in which all undifferentiated cells have the same tumourigenic capacity [101–103]. The heterogeneity of lung tumours suggests that they may follow a cancer stem cell model, but only a functional assay will definitely prove this hypothesis [104].

In human lung, CD133 was first suggested as a marker of cancer stem cells [105–107]. A small proportion of CD133^+^ cells were observed in primary SCLC and NSCLC, and were shown to have higher sphere-forming capacity *in vitro* than CD133^−^ cells [106]. Freshly isolated CD133^+^ cells from NSCLC have a higher tumourigenic potential than CD133^−^ cells after subcutaneous transplantation in immunocompromised animals and had self-renewal properties [106–108]. Treatment of the xenografted mice with cisplatin resulted in reduction of the tumour burden although CD133^+^ cells remained, suggesting that CD133^+^ cells may be a population of cancer stem cells resistant to standard chemotherapy [107]. More recently, CD166 was found to enrich for tumour-propagating cells in human lung adenocarcinomas. Transplantation of CD166-positive cells in immuno-compromised mice gave rise to tumours that recapitulated the heterogeneity of the primary tumour [109]. Increased metabolic activity in the glycine/serine metabolism enzyme pathway was observed in CD166^+^ cells and shown to induce oncogenesis [109]. Significantly, NSCLC patients with high expression of glycine decarboxylase, a glycine/serine metabolism enzyme overexpressed in CD166^+^ tumour cells, had the worst survival prognosis [109]. It is unclear whether CD133 and CD166 mark the same population of tumour-propagating cells. Genes that regulate cancer stem cell activity are still underexplored. It was suggested that the stem cell gene Oct-4 may be an important regulator of the cancer stem cell properties of CD133-positive cells [108].

In mouse models, Sca-1 appears to segregate distinct tumour-propagating cells in some mouse models of lung adenocarcinomas, but not in others [110]. Sca-1^+^ cells were enriched in cancer-propagating cells in K-Ras^G12D^p53^f/f^ tumours, but not in K-Ras^G12D^ mice. Conversely, only Sca-1^−^ cells had tumour-propagating activity in EGFR^L858R^ mouse model [110]. Genetic mutation status of mouse lung cancer therefore appears to change the phenotype of the tumour-propagating cells. It remains to be evaluated whether, as in the mouse, markers of cancer stem cells in human lung tumours differ according to the genetic mutation status of individual tumours.

## 8. Conclusion

Works in other tumour types have highlighted the importance of dissecting the cellular hierarchy in normal tissue in order to understand potential cells of origin in cancers [87,88]. In the lung, prospective isolation of lung stem and progenitor cells has been hindered by the lack of *in vivo* repopulation stem cell assays. Bioengineering strategies to develop a decellularized rodent lung matrix bioreactor system in which a lung scaffold is concurrently seeded with microvasculature cells and connected to a ventilation system could potentially constitute a surrogate assay to evaluate adult lung stem cell function [111–113]. A similar strategy was recently used to demonstrate that lung progenitor cells derived from embryonic stem cells could repopulate a decellularized lung [114]. Until now, *in vitro* culture of sorted cell populations and lineage-tracing strategies have provided some insights into the organization of the mouse lung and the identification of progenitor cells. Prospective isolation of these cells in human and in genetically engineered mouse models will enable the dissection of molecular mechanisms regulating self-renewal and differentiation at steady state and in lung repair after injury.

The cell of origin of most cancers remains unknown. Genetically defined mouse models of lung cancer have given insights into the possible cell of origin of K-ras^G12D^-induced adenocarcinomas and SCLC (Rb1/p53 loss) (figure 2). Other oncogenic driver mutations have been discovered in NSCLC, although at a lower frequency than K-ras mutations [115–120]. Development of mouse models in which these genetic transformations occur as well as use of genetically characterized human lung tumours will allow a better understanding of the contribution of oncogenic driver mutations and cells of origin to lung tumour heterogeneity [8]. Establishing a link between the first cell in which a specific mutation occurs and the molecular subtypes of lung cancer will enable better stratification of patients for improved therapeutic strategies.
